# Reversible frontotemporal brain sagging syndrome

**DOI:** 10.1212/WNL.0000000000001898

**Published:** 2015-09-01

**Authors:** Catherine F. Slattery, Ian B. Malone, Shona L. Clegg, Jason D. Warren, Nick C. Fox

**Affiliations:** From UCL Institute of Neurology, London, UK.

A 71-year-old man presented with 6 years of forgetfulness, behavioral change, intrusive “growling” vocalizations, orthostatic headaches, and a cough. MRI brain was consistent with frontotemporal brain sagging syndrome (figure, A). He subsequently fell, hitting his chest on a chair, with immediate resolution of his cough, cognitive improvement, and corresponding radiologic desagging (figure, B; video on the *Neurology*® Web site at Neurology.org).

**Figure:**
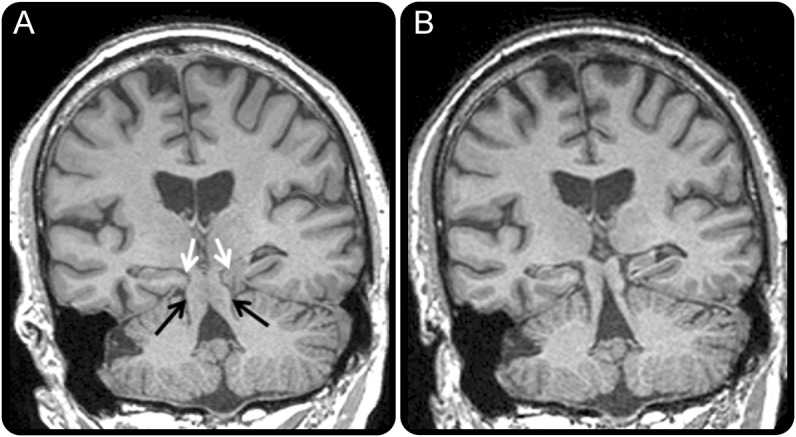
Coronal volumetric T1 MRI brain appearances of brain sagging and its resolution MRI at presentation (A) shows midbrain descent below the tentorium (black arrows) and posterior parahippocampal and lingual gyri herniation (white arrows). Follow-up MRI (B) demonstrates restoration of normal brainstem and medial temporal lobe configuration.

Frontotemporal brain sagging syndrome may be caused by intracranial hypotension secondary to CSF leakage along nerve root sleeves and is a potentially treatable frontotemporal dementia mimic.^1^ In this case, the fall may have caused a contusion injury and given him an auto-blood patch.

## Supplementary Material

Data Supplement
