# Supplementary material for: Reversible frontotemporal brain sagging syndrome
Source: Neurology. 2015 Sep 1;85(9):833. doi: 10.1212/WNL.0000000000001898 (PMC4553025; doi:10.1212/WNL.0000000000001898)
Supplement: Data Supplement [file supp_85_9_833__index.html]

Data Supplement 

# Reversible frontotemporal brain sagging syndrome

## Data Supplement

One video and legend; one .mp4 file and one Microsoft Word file.

**Neurology® data supplements are not copyedited before publication. Published editorials and translations have been copyedited.  
 © 2015 American Academy of Neurology.  
  
 Files in this Data Supplement:**

- Video e-1 - .mp4 file
- Video e-1 legend - Microsoft Word file
