# Supplementary material for: Reversible frontotemporal brain sagging syndrome
Source: Neurology. 2015 Sep 1;85(9):833. doi: 10.1212/WNL.0000000000001898 (PMC4553025; doi:10.1212/WNL.0000000000001898)
Supplement: Data Supplement [file supp_WNL.0000000000001898_Video_e-1_legend.docx]

**Video Legend**

Non-linear registration of MRI scans before and after symptom resolution, demonstrating restoration of normal anatomy from the previous herniated configuration.

After brain segmentation, the baseline scan was non-linearly registered[^1^](#_ENREF_1) to the repeat scan. The final displacement field was progressively scaled and applied to the baseline image to generate animation frames.

1. Modat M, Ridgway GR, Taylor ZA, et al. Fast free-form deformation using graphics processing units. Comput Methods Programs Biomed 2010;98:278-284.
